# Regional access to a centralized extracorporeal membrane oxygenation (ECMO) service in Victoria, Australia

**DOI:** 10.1016/j.ccrj.2023.11.007

**Published:** 2023-12-13

**Authors:** Joanna WY. Chow, John F. Dyett, Steve Hirth, Julia Hart, Graeme J. Duke

**Affiliations:** a Box Hill Hospital, Eastern Health, VIC, Australia; b Alfred Hospital, Alfred Health, VIC, Australia; c Australian and New Zealand Intensive Care Research Centre, Monash University, VIC, Australia; d Monash Eastern Clinical School, VIC, Australia

**Keywords:** Administration and health services, Extracorporeal life support, Intensive care, Cardiac failure, Resuscitation

## Abstract

**Introduction:**

Victoria, Australia provides a centralised state ECMO service, supported by ambulance retrieval. Equity of access to this service has not been previously described.

**Objective:**

Describe the characteristics of ECMO recipients and quantify geographical and socioeconomic influence on access.

**Design:**

Retrospective observational study with spatial mapping.

**Participants and setting:**

Adult (≥18 years) ECMO recipients from July 2016–June 2022. Data from administrative Victorian Admissions Episodes Database analysed in conjunction with Australian Urban Research Infrastructure Network population data and choropleth mapping. Presumed ECMO modes were inferred from cardiopulmonary bypass and pre-hospital cardiac arrest codes. Spatial autoregressive models including Moran's test used for spatial lag testing.

**Outcomes:**

Demographics and outcomes of ECMO recipients; ECMO incidence by patient residence (Statistical-Area Level 2, SA-2) and Index of Relative Socioeconomic Advantage and Disadvantage (IRSAD); and ECMO utilisation adjusted for patient factors and linear distance from the central ECMO referral site.

**Results:**

631 adults received ECMO over 6 years, after exclusion of paediatric (n = 242), duplicate (n = 135), and interstate or incomplete (n = 72) records. Mean age was 51.8 years, and 68.8 % were male. Overall ECMO incidence was 3.00 ± 3.95 per 10^5^ population. 135 (21.4 %) were presumed VA-ECMO, 59 (9.3 %) presumed ECPR, and 437 (69.3 %) presumed VV-ECMO. Spatial lag was non-significant after adjusting for patient characteristics. Distance from the central referral site (dy/dx = 0.19, 95% CI −0.41–0.04, p = 0.105) and IRSAD score (dy/dx = 0.17, 95% CI −0.19–0.53, p = 0.359) did not predict ECMO utilisation.

**Conclusion:**

Victorian ECMO incidence rates were low. We did not find evidence of inequity of access to ECMO irrespective of regional area or socioeconomic status.

## 1 Introduction

Equity of community access to centralised low-volume highly specialised healthcare services is desirable but complex to achieve and can be measured in a variety of ways.^1^ Investigation of geographical and socioeconomic factors on access to healthcare services and resultant outcomes within Australia have demonstrated mixed results, often disproportionately affecting rural residents.[2], [3], [4], [5], [6] Extracorporeal membrane oxygenation (ECMO) is a form of advanced mechanical organ support for refractory cardio-respiratory failure.^7^ It is a low-volume, complex, and time-critical intervention, with a volume–outcome relationship showing improved survival.[7], [8], [9], [10]

The State of Victoria, Australia, covering 227,444 km^2^, has a population of 6.7 million concentrated mainly in the central (68 %) and outer (19 %) urban areas of Melbourne.^11^ The state's ECMO service includes several credentialled Intensive Care Units (ICUs) operating within a tiered model governed by the Victorian state ECMO Service (VECMOS) and Safer Care Victoria.^7^ VECMOS provides a centralized service with one adult and one paediatric primary referral and retrieval site, plus credentialled intermediate and initiation sites, working in conjunction with the state-wide ambulance retrieval service.^12^ Of note, all but one of these sites are in metropolitan Melbourne and comprise only 5 % (8 out of 154) of all public health services in the state.[13], [14], [15]

There are several well-described statistical methods to evaluate geographic access to, and utilisation of health services.^1^^,^^16^^,^^17^ Choropleth maps are a common method of visually displaying data trends across a geographic region.^18^ Spatial autoregression models permit analysis of geographical factors when healthcare access is subject to spatial lag, where an outcome variable in a location is influenced by both it and its neighbours’ independent and dependent variables.^19^ Evaluating the spatial distribution of disease and access to ECMO services is therefore of clinical interest and useful for health service planning. As access to ECMO in the state is provided by an established retrieval service and a state-wide referral pathway, we hypothesised that access is equitable across all geographic locations in Victoria. The aims of the study were therefore to describe the 1) characteristics of adult ECMO recipients, and 2) equity of access, using spatial autoregressive models and choropleth mapping.

## 2 Methodology

### 2.1 Study design

We conducted a retrospective observational study of all consecutive adults who received ECMO between 1st July 2016 and 30th June 2022. Approval was granted by the Eastern Health Office of Ethics and Research (LR19/047), classified as low-risk, and the need for individual patient consent was waived.

### 2.2 Data sources

Three data sources were linked for this analysis. The Victorian Admitted Episodes Dataset (VAED) is an administrative database of all hospital separations and provides patient demographic information, diagnoses, outcomes, and procedures, including ECMO. The Australian Bureau of Statistics provided estimates of annual age- and sex-matched population based on census data. The open-source Geographical Information System (Q-GIS) platforms of the Australian Urban Research Infrastructure Network (AURIN)^20^ contain datasets and tools for choropleth mapping.

### 2.3 Study setting

From the VAED, we identified all adult (age ≥18 years) hospital separations associated with an ECMO procedure code over the 6-year study period. We recorded the place of residence, indicated by Statistical Area Level-2 (SA-2), an Australian standard geographical classification system (smaller than local government and postcode areas) closely associated with community socioeconomic variables. Locality was also mapped by Local Government Area (LGA) to calculate the direct distance to the central referral site (Alfred Hospital). All VECMOS retrievals are transferred to the Alfred, including cases requiring transfer from credentialled Intermediate and Initiation sites, particularly for VV-ECMO.^12^ All but one of these ECMO sites are within a 25 km radius of the Alfred. We extracted demographic data (age, sex, year of separation, source of admission), diagnostic codes (admission diagnoses, cardiac arrest, comorbidities, COVID-19 status), procedure codes (mechanical ventilation, renal replacement therapy, cardiac surgery, cardiopulmonary bypass) and outcome data (ICU and hospital length of stay, and hospital discharge status). Comorbidity burden was calculated using the Elixhauser method.^21^

To account for duplicate records, we excluded sending-hospital episodes and only analysed final hospital episodes. The VAED procedure codes do not identify the mode of ECMO - Veno-Venous ECMO (VV-ECMO), Veno-Arterial ECMO (VA-ECMO), or ECMO-Cardiopulmonary Resuscitation (ECPR). We applied *a priori* rules to define three surrogate subgroups: ECMO recipients who also received cardiopulmonary bypass as surrogate for presumed VA-ECMO; out-of-hospital cardiac arrest not coded for CPB as surrogate for presumed ECPR; and those without OOHCA or CPB codes, as surrogate for presumed VV-ECMO. We use the qualifier “presumed” to improve readability whilst acknowledging the limitations of the data.

### 2.4 Outcome measures

Primary outcomes included the ECMO incidence, hospital length of stay and outcome. Secondary outcomes included regional ECMO utilisation rate, defined as number of ECMO cases per 100,000 age-matched population, by patient residential origin, and ECMO utilisation rate adjusted for distance from the central ECMO site, socioeconomic status, and patient factors such as admission diagnoses, comorbidities, and procedures. ECMO utilisation rate by avoidable cardiovascular mortality rate for each LGA was also mapped.

### 2.5 Geographic mapping

We obtained the Index of Relative Socio-Economic Advantage and Disadvantage (IRSAD) and rates of avoidable cardiovascular deaths for each region from AURIN. The IRSAD score is a composite measure reflecting the socioeconomic well-being of a geographical area, where higher scores reflect more socio-economically advantaged areas.^22^ The Q-GIS choropleth map tool was used to spatially map ECMO utilisation rates as colour-coded circles overlying IRSAD scores or avoidable cardiovascular deaths by LGA.

### 2.6 Statistical analysis

Results are presented using mean (standard deviation, SD), median (interquartile range, IQR), or frequency counts (percentages) where appropriate. ECMO incident rates were displayed as incidence per 100,000 population and is aggregated over the study period. For the population rate (denominator), we used the 2018 ABS population number. Using the StataMP™ v17 (2019, College Station, TX) statistical software command *spregress,* spatial autoregression models were fitted to ECMO utilisation rates for each SA-2. The command *impact* was used to estimate the margins. The form of the estimator was

*spregress ecmo distance metro pop patient, gs2, dvarlag*

where ecmo = ECMO therapy, distance = travel distance to the CSP, metro = metropolitan or rural resident; population = SA-2 population for 2018, patient = patient factors (see below), gs2 = generalized spatial two-stage least-squares estimator; and dvarlag = the spatial weighting matrix derived from ABS data, defined by the spatial lag of the place of residence.

Models were tested with and without adjustment for patient factors: demographics, mean IRSAD score, comorbidities, admission diagnoses, type of cardiac surgery, and season (year and month of admission). Errors were assumed to be heteroskedastic. Spatial lag of ECMO utilisation was assessed using Moran's test, an estimate of residual correlation. Univariate and multivariate marginal effects of the covariates on the ECMO utilisation rate were performed. Sensitivity analyses were performed on the subgroups. The interaction between subgroups, ECMO utilisation, and IRSAD and avoidable deaths were assessed using logistic regression. A p-value of <0.05 was accepted as significant.

## 3 Results

### 3.1 Study population

**Fig. 1:**
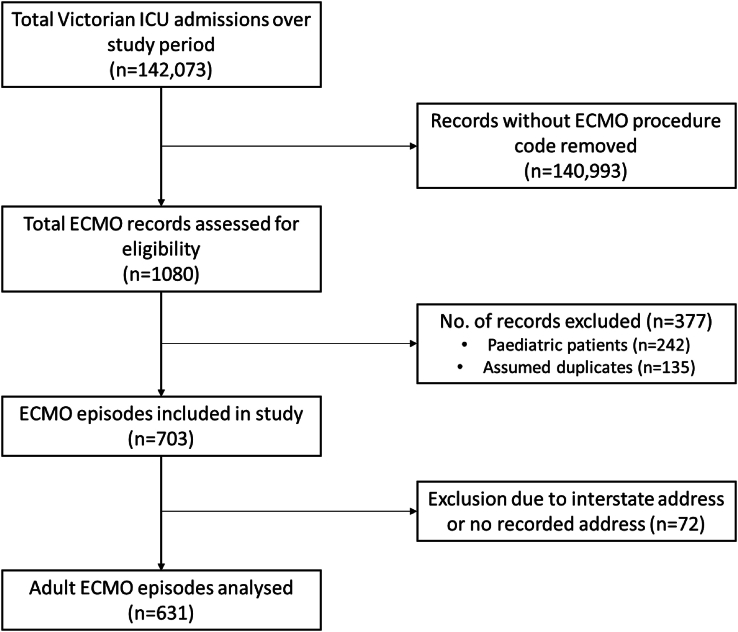
Study flowchart of enrolment of Victorian adult ECMO recipients. ECMO, Extracorporeal Membrane Oxygenation.

There were a total of 1080 VAED ECMO episodes identified over the 6-year study period. After excluding paediatric patients (n = 242), duplicates (n = 135), and those from interstate or no recorded address (n = 72), there were 631 adult ECMO records for analysis (Fig. 1). Estimated completeness of the SA-2 data was at least 93 %, with missing data originating from private sector hospital records.

The mean age of the study population was 51.8 years (±14.4 years) with male preponderance (68.8 %; Table 1). Baseline characteristics, procedures, and outcomes are displayed in Table 1. Baseline characteristics of metropolitan versus regional or rural ECMO recipients are displayed in Appendix Table S1. There were 135 presumed VA-ECMO (21.4 %) episodes, 59 (9.3 %) presumed E-CPR episodes and 437 (69.3 %) presumed VV-ECMO episodes, including 63 (10 %) with COVID-19 infection. Overall hospital survival was 50 % (n = 315; 95% CI = 45–56 %) and 45 %, 54 %, and 34 % for presumed VA-ECMO, VV-ECMO, and ECPR, respectively.

**Table 1:** Patient characteristics of adult ECMO recipients in Victorian ICUs between July 2016 and June 2022.

Characteristics	All patients (n = 631)	Subgroups		
		Presumed VA-ECMO (n = 135) 21.4 %	Presumed VV-ECMO (n = 437) 69.3 %	Presumed ECPR (n = 59) 9.3 %
Age (years), mean (SD)	51.8 (14.4)	59.7 (13.9)	50 (13.8)	46.6 (13.3)
Male, n (%)	434 (68.8)	100 (74.1)	297 (68)	37 (62.7)
Emergency admission, n (%)	546 (86.5)	88 (65.2)	400 (91.5)	58 (98.3)
**Diagnoses:**				
Sepsis, n (%)	257 (40.7)	31 (23)	212 (48.5)	14 (23.7)
Acute kidney injury, n (%)	202 (32)	22 (16.3)	162 (37.1)	18 (30.5)
Circulatory shock, n (%)	199 (31.5)	23 (17)	158 (36.2)	18 (30.5)
Cardiogenic shock n (%)	130 (20.6)	16 (11.9)	99 (22.7)	15 (25.4)
Bacterial community acquired pneumonia, n (%)	105 (16.6)	9 (6.7)	89 (20.4)	7 (11.9)
COVID-19, n (%)	63 (10)	0 (0)	62 (14.2)	1 (1.7)
Liver failure, n (%)	39 (6.2)	3 (2.2)	33 (7.6)	3 (5.1)
Septic shock, n (%)	38 (6)	2 (1.5)	36 (8.2)	0 (0)
**Comorbidities:**				
Congestive cardiac failure, n (%)	265 (42)	88 (65.2s)	158 (36.2)	19 (32.2)
aFrail, n (%)	176 (27.9)	45 (33.3)	122 (27.9)	9 (15.3)
Chronic liver disease, n (%)	96 (15.2)	25 (18.5)	64 (14.6)	7 (11.9)
Chronic kidney disease, n (%)	49 (7.8)	26 (19.3)	19 (4.3)	4 (6.8)
Diabetes, n (%)	122 (19.3)	40 (29.6)	78 (17.8)	4 (6.8)
Any cancer, n (%)	41 (6.5)	8 (5.9)	31 (7.1)	2 (3.4)
COPD, n (%)	36 (5.7)	11 (8.1)	24 (5.5)	1 (1.7)
Metastatic solid tumours, n (%)	6 (1)	2 (1.5)	4 (0.9)	0 (0)
Elixhauser comorbidity score, median (IQR)	3 (2–5)	6 (4–10)	7 (4–15.25)	2 (3–6)
**Interventions and outcomes:**				
Hospital mortality, n (%)	316 (50.1)	74 (54.8)	200 (45.8)	42 (71.2)
ICU LOS (days), mean (SD)	18.6 (23.7)	15.9 (15.1)	20.8 (26.4)	9.2 (13.8)
Hospital LOS (days), mean (SD)	26.7 (30.4)	27.6 (24.9)	28.3 (32.7)	12.7 (17.6)
Duration mechanical ventilation (days), mean (SD)	14.9 (35.7)	11.4 (11.7)	17 (23.5)	7.2 (10)
Renal Replacement Therapy, n (%)	305 (48.3)	72 (53.3)	208 (47.6)	25 (42.4)
Percutaneous Coronary Intervention, n (%)	81 (12.8)	13 (9.6)	52 (11.9)	16 (27.1)
Coronary Artery Graft Surgery, n (%)	82 (13)	77 (57)	5 (1.1)	0 (0)
Valvular surgery, n (%)	61 (9.7)	60 (44.4)	1 (0.2)	0 (0)
Ventricular Assist Device, n (%)	4 (0.6)	4 (3)	0 (0)	0 (0)
Heart transplant, n (%)	15 (2.4)	8 (5.9)	7 (1.6)	0 (0)

Subgroups: Presumed VA-ECMO were those who received cardiopulmonary bypass, presumed VV-ECMO were those did not receive cardiopulmonary bypass and did not have an admission diagnosis of out-of-hospital cardiac arrest, ECPR were those who did not receive cardiopulmonary bypass and had an admission diagnosis of out-of-hospital cardiac arrest.

ECMO, Extracorporeal membrane oxygenation; VA-, Venoarterial-; VV, Venovenous-; ECPR, Extracorporeal Cardiopulmonary Resuscitation; COVID-19, Coronavirus-19 disease; COPD, Chronic Obstructive Pulmonary Disease; LOS, length of stay.

a The frail score was based on the modified Hospital Frailty Risk Score, which comprises ICD-10 diagnostic codes.^38^

**Fig. 2:**
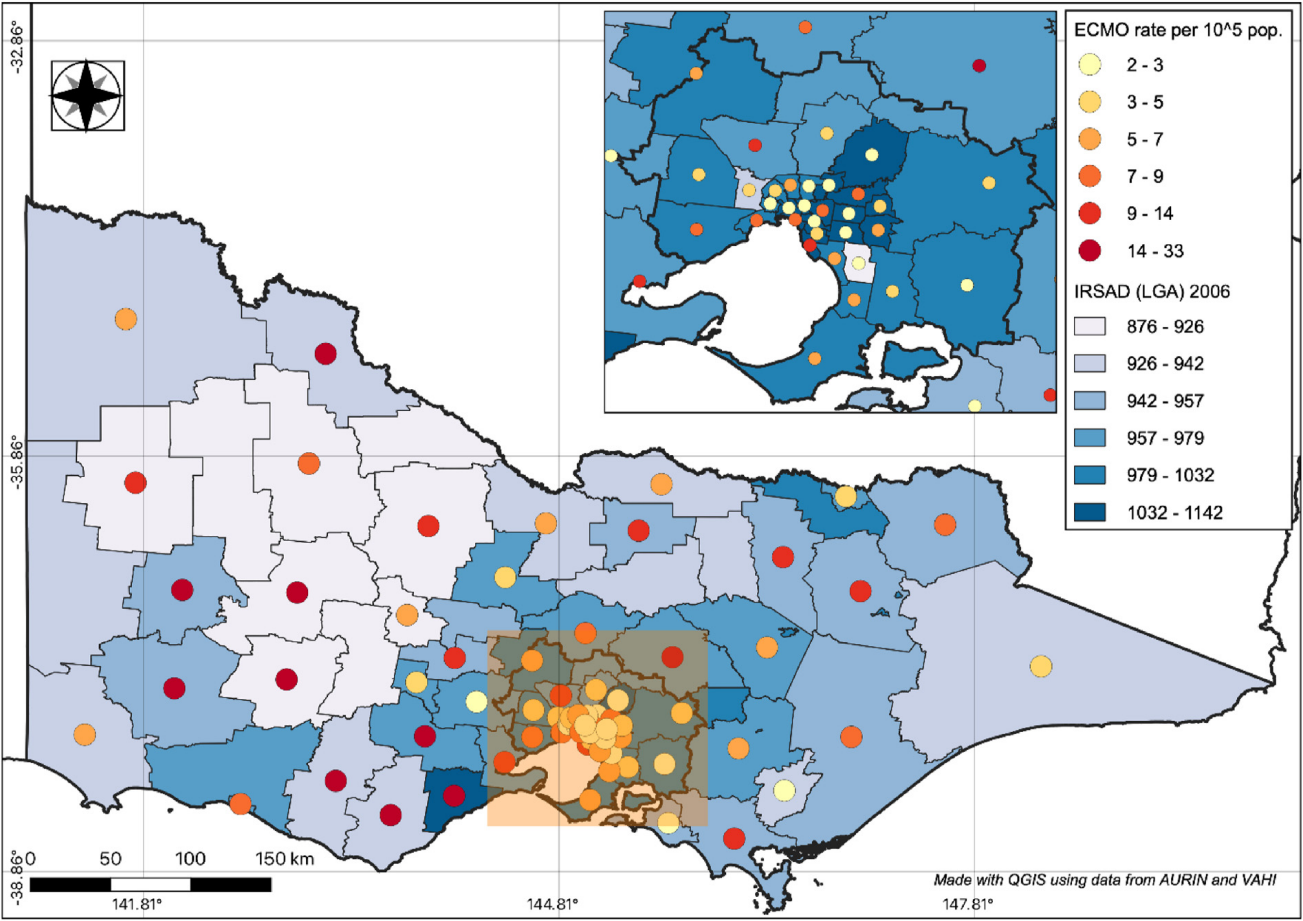
Choropleth map of crude ECMO utilisation rates overlying socioeconomic status (IRSAD scores) for LGAs in the state of Victoria. Inset: Metropolitan Melbourne area. ECMO utilisation rate per 100,000 population over 6 years are displayed as circles, with increasing colour intensity representing higher utilisation rates. LGAs with no recorded ECMO utilisation do not have an overlying circle. LGAs with increasingly darker blue-coloured background shades correspond with higher mean IRSAD scores and therefore more socio-economically advantaged areas. ECMO, Extracorporeal membrane oxygenation; LGA, Local Government Area; IRSAD, Index of Socioeconomic Advantage and Disadvantage.

The overall ECMO utilisation rate was 3.00 ± 3.95 per 10^5^ (18 ± 23.7 per 10^5^ over 6 years) age-matched population, ranging between 0 and 28 per 10^5^ population across SA2 regions. The presumed VA-ECMO, VV-ECMO, and ECPR ECMO utilisation rate was 0.8, 2.2, and 0.4 per 10^5^ population, respectively. The choropleth map of ECMO rate over 6 years for each LGA, against IRSAD scores and avoidable cardiovascular death rate, is displayed in Fig. 2 and Fig. S1, respectively.

Moran's test for spatial lag of ECMO utilisation rate based on the direct distance of the referral site to the Alfred Hospital was not statistically significant (χ^2^ = 0.47, p = 0.49). In the univariate analysis in spatial autoregression modelling, ECMO utilisation appeared to be associated with proximity to the Alfred Hospital, increasing IRSAD score, COVID-19 diagnosis, male gender, and higher comorbidity score (Table 2). After adjusting for SA-2 population size, ECMO utilisation rate was not statistically significantly affected by distance from the Alfred Hospital (distance per 100 km dy/dx = 0.19, 95% CI −0.41–0.04, p = 0.105) and IRSAD score. Comorbidity and male sex retained significant multivariate associations with ECMO utilisation. Results were unchanged when adjusted for the subgroups, COVID-19 infection, year of ECMO use, diagnosis or procedure.

**Table 2:** Multivariate analysis results of statistically significant covariates in univariate marginal effects analysis on ECMO utilisation rate.

Covariate	Univariate dy/dx^a^ (95% CI)	P-value	Multivariate dy/dx^a^ (95% CI)	P-value
Distance (per 100 km)	−2.63 (−3.61, −1.66)	<0.001	−0.19 (−0.41, 0.04)	0.105
IRSAD (per 100 points)	4.01 (1.78, 6.24)	<0.001	0.17 (−0.19, 0.53)	0.359
Comorbidity	0.26 (0.25, 0.27)	<0.001	0.15 (0.12, 0.17	<0.001
Male	1.37 (1.30, 1.44)	<0.001	0.60 (0.45, 0.75)	<0.001
Mean age (per year)	0.11 (0.04, 0.18)	0.003	0.01 (−0.39, 0.57)	0.888
COVID-19 diagnosis	4.61 (1.58, 7.65)	0.003	0.40 (−0.06, 0.86)	0.092

IRSAD, Index of Relative Socioeconomic Advantage and Disadvantage; COVID-19, Coronavirus disease.

a dy/dx = change in outcome for each unit change in covariate. A positive number (>0) implies increased ECMO utilisation rate and a negative number (<0) implies decreased ECMO utilisation rate. Marginal effects based on the Stata command estat impact are not to be confused with regression coefficients. Only covariates with statistically significant effects on the ECMO utilisation rate in the univariate analysis are reported.

## 4 Discussion

### 4.1 Key findings

We undertook a retrospective observational study on geospatial ECMO utilisation, and did not find evidence to suggest inequity of access to ECMO in the state of Victoria. Importantly, the distance from the central ECMO referral centre and the patient's socioeconomic status did not appear to be associated with utilisation of ECMO. There was also no difference between presumed modes of ECMO, or patients with or without COVID.

### 4.2 Relationship to other studies

The Victorian ECMO incidence rate of 3.0 per 10^5^ was comparable to Germany's 3.5 per 10^5^ (2011–2014),^23^ whereas in China in 2018 this was 0.148 per 10^5^ population.^24^ Our study's ECMO rate for COVID-19 patients (2.5 per 100,000) was similar to a multicentre cohort study in greater Paris,^25^ whilst a Chilean study with 94 patients reported an incidence of 0.42 per 10^5^ population.^26^ The differences demonstrate the difficulties in comparing different healthcare systems, therefore making a strong argument to undertake analysis of local data where results may be quite different.

Several observational studies report regional, seasonal, or socioeconomic differences in ECMO incidence rates.^24^^,^^27^^,^^28^ A USA cross-sectional study reported between 59 and 96 % of their adult population have geographic access to any ECMO centre.^29^ Their wide incidence range is likely due to differing service models, definition of “geographic access”, and availability of interhospital transport services. In China, less developed regions had lower ECMO utilisation rates,^24^ and in the USA, ECMO recipients were more likely to reside in higher income neighbourhoods.^28^ In addition, the COVID-19 pandemic disproportionately impacted racial groups and those from socioeconomically disadvantaged backgrounds,^30^ with a statistically significant increase in mortality in those who were eligible but did not receive ECMO due to limited resources.^31^ Conversely, geography and socioeconomic status did not appear to impact the ECMO utilisation rate in our COVID-19 subgroup, in part due to the lower incidence of COVID-19 in Australia. The marginal effects analysis also did not identify a seasonal effect on the ECMO utilisation rate.

The overall survival to hospital discharge in this study was 50 %, consistent with the international Extracorporeal Life Support Organization (ELSO) registry's reported 52 % survival.^32^ The study population mean age and male preponderance is similar to published reports.[33], [34], [35] Patients who required transfer to the referral centre had a favourable outcome (82 % hospital survival). An earlier New South Wales study of patients transferred on ECMO reported a 68 % hospital survival rate.^36^

### 4.3 Implications of study findings

In those who received ECMO in Victoria, we did not find evidence of inequity of access to ECMO, irrespective of socioeconomic status or geographical location. We used multiple adjusters in the SAR models and found that ECMO utilisation was not associated with distance from the Alfred Hospital, socioeconomic status, or COVID-19 diagnosis. This could reflect the coordination between a centralised service and retrieval service in a relatively smaller state with fewer remote areas.

### 4.4 Strengths and limitations

To our best knowledge, this is the first study depicting not just how, but also where ECMO utilisation in Victoria is by means of visual geographic representation on a heat map. We differentiated CPB (presumed VA-ECMO) from non-CPB (presumed VV-ECMO and ECPR) codes given they are conceptually different supports with different patient selection, survival rates, and techniques, which are important considerations when designing a state-wide service delivery. We were able to demonstrate the use of novel methodology, commonly employed in geospatial research, to describe access to a time-critical service where inequity might be reasonably expected. It can therefore be replicated in other states and territories, and for other pathologies.

This study has several limitations. First, regional rates of ECMO utilisation were low resulting in wide confidence intervals. A Type II error therefore cannot be excluded. This in part could explain why other time-critical services, such as for acute stroke, have found regional differences,^37^ whereas we have not. The presumed ECPR subgroup had very small numbers per region, and we could have missed a significant result in this subgroup. Other methodologies (like a web-referral system) investigating the influence of geography on access to ECPR are preferred. Ability to access ECMO at six other credentialled sites to a limited degree could have confounded our results. Nonetheless, all but one site is in Metropolitan Melbourne and is unlikely to have significantly impacted regional access.

Moran's is an unadjusted test for spatial lag, and can ineffectively account for a heterogenous population, therefore requiring a larger sample size. We extended the study over 6 years to help reduce but not eliminate this risk. A larger study requires a longer time and introduces temporal sources of bias. Socioeconomic status, avoidable death rate and population numbers also change over time, limiting the ability to perform longitudinal analysis. We accounted for temporal effects, especially from the COVID-19 pandemic, by adjusting for calendar year of admission, and found that this was not a significant confounder. Geographic origin was missing for 7 % of participants, who were excluded. Their inclusion is unlikely to have changed our results.

The VAED do not distinguish modes of ECMO and we present one way of deriving presumed modes, which may under- or overestimate true numbers.^7^ The VAED also does not capture the temporal sequence or case complexity. ECMO recipients are a highly selected group. This might be expected to increase disparity in geographic access to ECMO. We were however only able to answer the question of whether there was inequity of access in those who received ECMO. Further studies are required to investigate if there is equity of access for those who meet clinical eligibility criteria, and resultant outcomes, in addition to the underlying disease patterns in the regional population.

## 5 Conclusion

The incidence of ECMO utilisation by adult residents in Victoria is approximately 3.0 per 100,000 per annum. We did not find evidence to suggest inequity of access to the ECMO service in this state, irrespective of distance from the central service provider or socioeconomic status. Analysis of health service access using visual mapping and statistical tools that adjust for geographic, socioeconomic, and other patient factors appear feasible and worthy of application to other acute health services.

## Conflict of interest

The authors declare that they have no known competing financial interests or personal relationships that could have appeared to influence the work reported in this paper.

## Ethical approval

Approval was granted by the Eastern Health Office of Ethics and Research (LR19/047), classified as low-risk, and the need for individual patient consent was waived.

## Author contributions

Dr J Chow, Dr G Duke, Dr J Dyett, Dr J Hart, and Mr S Hirth contributed to the conception of design, drafting, revision, and final approval to be published. Dr J Chow, Dr G Duke, Dr J Dyett contributed to the manuscript preparation and data interpretation. In addition, Dr J Dyett contributed to the visual mapping analysis and Dr G Duke contributed to the data extraction and statistical analysis.
