# Supplementary material for: Regional access to a centralized extracorporeal membrane oxygenation (ECMO) service in Victoria, Australia
Source: Crit Care Resusc. 2023 Dec 13;26(1):47–53. doi: 10.1016/j.ccrj.2023.11.007 (PMC11056431; doi:10.1016/j.ccrj.2023.11.007)
Supplement: Multimedia component 1 [file mmc1.docx]

This appendix was part of the submitted manuscript and has been peer reviewed. It is posted as supplied by the authors.

| **Appendix Table S1** Baseline characteristics of metropolitan versus rural or regional ECMO recipients in Victoria. | | |
| --- | --- | --- |
|  | Metropolitan | Rural/Regional |
| Number of patients, n (%) | 427 (68) | 204 (32) |
| Age, median (IQR) | 51 (41-61) | 56 (47-65) |
| Male, n (%) | 295 (69) | 138 (67) |
| Charlson score, mean (SD) | 1.44 (1.21) | 1.58 (1.23) |
| Indigenous, n (%) | 3 (0.7) | 5 (2.5) |
| COVID-19 infection, n (%) | 55 (13) | 8 (4) |
| Pre-hospital cardiac arrest, n (%) | 56 (13) | 19 (9) |
| Survived hospital stay, median (IQR) | 32 (17-56) | 35 (21-54) |
| Did not survive hospital stay, median (IQR) | 7 (2-17) | 5 (2-12) |
| In-hospital mortality, n (%) | 210 (49) | 104 (51) |
| *ECMO = Extracorporeal membrane oxygenation, COVID-19 = Coronavirus-19 disease.* | | |

**Appendix Figure S1** Choropleth map of crude ECMO utilisation rates overlying avoidable cardiovascular mortality rates for LGAs in the state of Victoria.

**
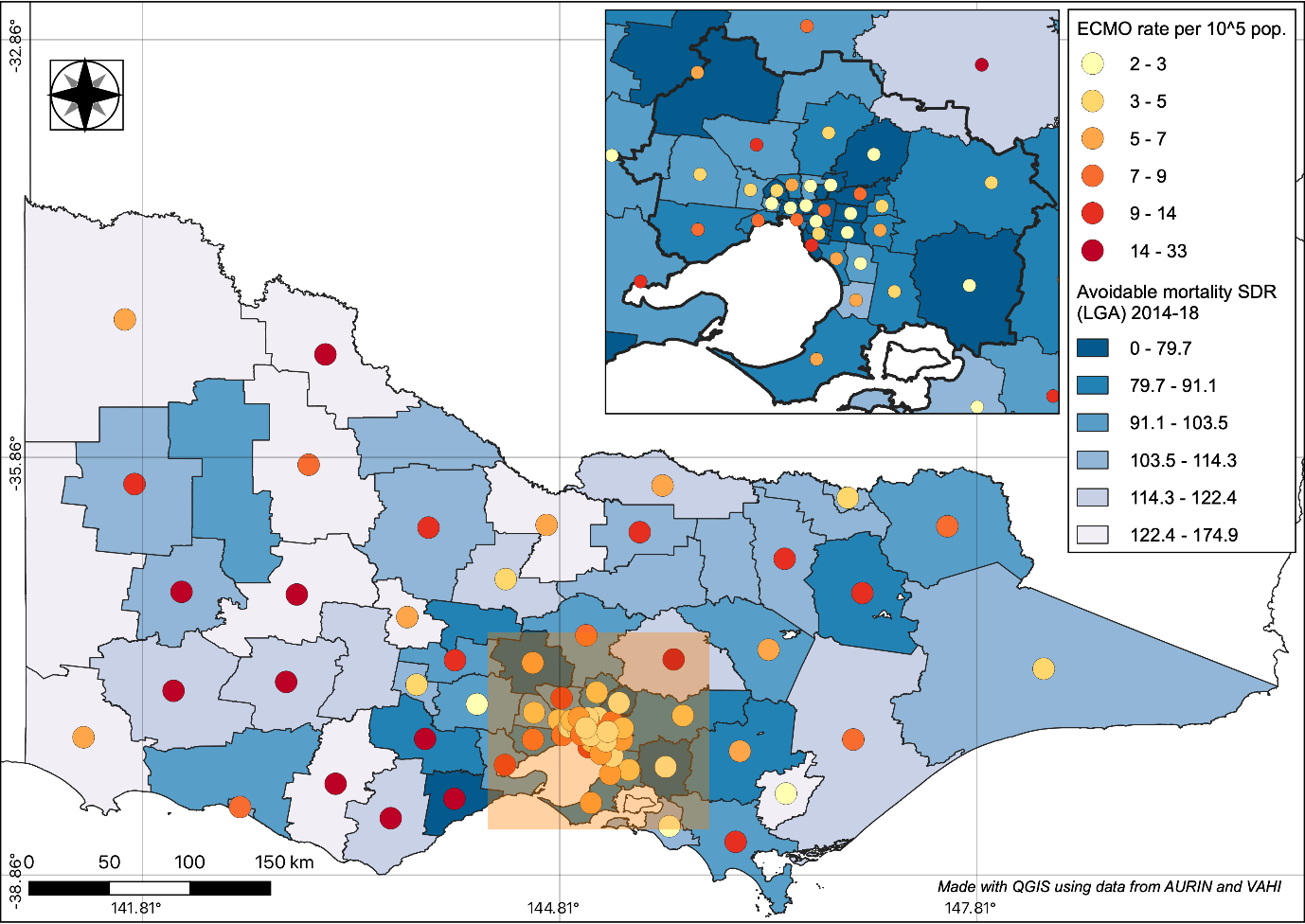
**

Inset: Metropolitan Melbourne area.

ECMO utilisation rate per 100,000 population over 6 years are displayed as circles, with increasing colour intensity representing higher utilisation rates. LGAs with no recorded ECMO utilisation do not have an overlying circle. LGAs with lighter blue-coloured background shades correspond with higher age-standardised cardiovascular avoidable mortality rates per 100,000 population.

ECMO = Extra-corporeal membrane oxygenation, LGA = Local Government Area, SDR = age-standardised death rate
